# Notice of a Case of Human Rumination, Where an Examination Was Made of the Body of the Subject after Death

**Published:** 1821-07

**Authors:** George Nesse Hill

**Affiliations:** Member of the Royal College of Surgeons, &c.


*Notice of a Case of Human Rumination, where an examination was made of the Body of the Subject after Death.* By George Nesse Hill, Member of the Royal College of Surgeons, &c.

AN interesting communication on the subject of human rumination appears in your Number for May, by Dr. Copland, containing some original and excellent remarks on this curious and rather uncommon malady: among others, Dr. C. observes, "the only instance upon record, to my knowledge, in which inspection after death took place, was in the instance of this affection occurring in a monk."

Enthusiasts in their arduous profession (which all young medical men ought to be,) are greatly indebted to such learned physicians as Dr. Copland, Dr. Cooke, &c. for their indefatigable researches into the early histories and records of the more ambiguous diseases; the utility of such condensed communications is greatly enhanced by the practical remarks accompanying them, as instanced in Dr. Copland's case of human rumination. This gentleman will not be displeased at my pointing out to him a case, and dissection, of similar disease in a severely-afflicted insane patient, who fell under my care in the year 1791; he appeared to fall a victim to epileptic insanity, the result of early indiscretion: that part of the history of this young man which related to his rumination of his food, bore an exact resemblance to the one so ably detailed by Dr. Copland. I examined the body in the presence of the venerable Haygarth, who, as well as myself, was much struck with the extreme tenuity and smooth internal surface of the stomach.

In order the more correctly to observe the ruminating process, I invited my patient to dinner several times; he eat with a ravenous appetite and wonderful quickness, but never finished a meal without commencing the ruminating process. A short history of this case will be found in the Appendix to my "Essay on the Prevention and Cure of Insanity."

*Chester, May*, 1821.

